# Optimal design for multi-arm multi-stage clinical trials

**DOI:** 10.1186/1745-6215-12-S1-A4

**Published:** 2011-12-13

**Authors:** James MS Wason, Thomas Jaki

**Affiliations:** 1 MRC Biostatistics Unit, Cambridge, CB2 0SR, UK; 2 Medical and Pharmaceutical Statistics Research Unit, Lancaster University, Lancaster, LA1 4YF, UK

## 

In early stages of drug development there is often uncertainty about the most promising among a set of different treatments. In order to ensure the best use of resources it is important to decide which, if any, of the treatments should be taken forward for further testing. Multi-arm multi-stage (MAMS) trials provide gains in efficiency over separate randomised trials of each treatment. They allow a shared control group, dropping of ineffective treatments before the end of the trial and stopping the trial early if sufficient evidence of a treatment being superior to control is found. Moreover a direct, head-to-head, comparison of treatments is undertaken that ensures that many potentially influential outside factors are eliminated.

In this talk we discuss optimal MAMS designs for normally distributed endpoints. An optimal design has the required type I error rate and power, but minimises the expected sample size (ESS) at some combination of treatment effects. Finding an optimal design requires searching over the stopping boundaries and sample size per stage, potentially a large number of parameters. We propose a method which combines quick evaluation of specific designs and an efficient stochastic search for the optimal design parameters. The search can also take the allocation ratio between controls and active treatments into account, allowing further efficiency gains.

In the two-arm case, the triangular design has excellent expected sample size properties, and is immediate to find. Here we find that there is potential for greater improvements over the triangular design, especially as the number of stages or active treatments increases. The triangular design still serves as a quick-to-find and near-optimal design, so may still be useful for design of MAMS trials.

